# MMR-deficiency and BRCA2/EGFR/NTRK mutations

**DOI:** 10.18632/aging.101275

**Published:** 2017-08-03

**Authors:** Prashanth Gokare, Amriti R. Lulla, Wafik S. El-Deiry

**Affiliations:** Department of Hematology/Oncology and Molecular Therapeutics Program, Laboratory of Translational Oncology and Experimental Cancer Therapeutics, Fox Chase Cancer Center, Philadelphia, PA 19111, USA

**Keywords:** MSI-H, CRC, BRCA2, EGFR, NTRK

Colorectal cancer (CRC) is the third most common cancer type in the developed world and a leading cause of cancer-related death. When detected at early stages, surgical interventions can lead to cure (92%, Stage I), and there is a better prognosis in Stages II and III as compared to stage IV [1]. Yet, molecular heterogeneity of tumors in early stage cancers may lead to variable clinical outcomes. This in general remains an area of active investigation in search of prognostic and predictive factors. There are some prognostic factors in stage II disease that confer high risk including tumor size, lymphovascular or perineural invasion, obstruction or perforation, and in stage III disease the spread to a majority of the lymph nodes examined is associated with worse outcomes.

While most of CRCs arise via genetic changes that turn on different driver genes; a small fraction of about ∼15% of cancers arise as a consequence of microsatellite instability (MSI) [2]. MSI-High (MSI-H) status of a CRC is the signature of a deficient or impaired DNA mismatch repair (dMMR) system in tumor cells and encompasses insertions or deletions in form short tandem repeats or “microsatellites” distributed in the genome as well as an increased mutation frequency. Most MSI-H CRC tumors arise due to the somatic inactivation of MLH1, MSH2, MSH6 and PSM2 genes. MSI-H CRCs also harbor mutations and genetic alterations in marker genes such as BRAF, MRE11A and TGF-β type II receptors [3] which may provide tailored treatment options for MSI-H patients, who otherwise poorly respond to adjuvant 5-FU chemotherapy.

In a recent study, we [4] identified BRCA2, EGFR and NRTK mutations to be strongly associated with the MSI-H phenotype in CRC. The novel findings shed light on potentially actionable therapeutic vulnera-bilities in MSI-H colorectal cancer.

Using a combination of bioinformatics and predictive modelling approaches, we showed that a large fraction (∼42%) of stage II MSI-H CRC patients have a higher frequency of BRCA2 mutations as compared to 6% of non-MSI-H patients. We characterized BRCA2 somatic mutations and found 75 unique mutations in 46% of MSI-H CRC patients, with the majority of the mutations being missense. Of note, the N-terminal and C-terminal of BRCA2 showed frequent mutations in the MSI-H CRC cohort. While C-terminal mutations affect the binding of DNA-damage response proteins such as Rad51, N-terminal mutations can render truncations in the BRCA2 protein. A key observation we made is the abundant coding microsatellite alterations in the BRCA2 gene, which could render the protein dysfunctional or truncated. This has therapeutic implications where MSI-H tumors could be potentially susceptible to PARP inhibitors if both BRCA2 alleles are disrupted within an MSI-H tumor. We anticipate that some MSI-H tumors may lose both BRCA2 alleles due to the finding of multiple BRCA2 mutations in some MSI-H tumors.

We also observed a high frequency of mutations in receptor tyrosine kinases (RTKs) such as EGFR and NTRK. 45% of the observed MSI-H CRC cases showed EGFR mutations, with a majority of these mutations in the TK i.e. kinase domain of EGFR. We did not observe enrichment of any particular EGFR mutation. However, most of the EGFR mutants were predicted to retain expression of their extracellular domain, making them actionable by existing antibody and small molecule therapies. Some of the mutations are known to be actionable by currently available first or later generation small molecule EGFR inhibitors. Given the enrichment of EGFR and BRAF mutations in MSI-H patients combinatorial therapies such as cetuximab and vemurafenib, targeting mutations in these two genes may provide enhanced efficacy. Lastly, we identify novel NTRK mutations in about 40% MSI-H CRC cases, with frequent mutations in the kinase domains of all three (NTRK1, NTRK2 and NTRK3) proteins. NTRK gene mutations are rare in cancer and can occur as gene fusions or point mutations resulting in increased kinase activity. The recent development of targeted agents like entrectenib and larotrectinib offer therapeutic options for patients whose tumors harbor actionable NTRK gene alterations. The study opens up new avenues of NTRK mutation targeting in MSI-H CRC patients who may be ideal candidates for such therapies.

In light of recent advancement of approval of anti-PD-1 (pembrolizumab) therapy for MSI-H tumors, the study by Deihimi et al., adds a novel facet to MSI-H CRC tumors biology for future exploration of combined immune and genomic based targeting, potentially extending to other MSI tumors in a tissue agnostic manner.
